# Social prescribing for frequent attenders in primary care: An economic analysis

**DOI:** 10.3389/fpubh.2022.902199

**Published:** 2022-10-14

**Authors:** Mary Lynch, Ceri R. Jones

**Affiliations:** ^1^School of Health and Life Sciences, University of West Scotland, Glasgow, United Kingdom; ^2^Department of Neuroscience, Psychology and Behaviour, University of Leicester, Leicester, United Kingdom

**Keywords:** social prescribing, economic analysis (EA), mental health, psychological wellbeing, primary care

## Abstract

**Background:**

Social prescribing (SP) is a mechanism to link patients with community groups and third sector organizations. It offers a complimentary approach to the traditional medical models to address psychosocial needs of patients more effectively and in turn aims to reduce demand on the NHS. The aim of this study was to explore the economic benefits related to changes in the use of healthcare resources following a social prescribing intervention in four primary care practices in Wales.

**Methods:**

Quantitative data from routine healthcare usage was collected from the 78 participants pre and post-intervention. The participants were grouped into frequent attenders (FA) (*n* = 21) and frequent (*n* = 57) non-attenders (FNA), and a cost analysis was conducted to estimate cost variances based on healthcare unit usage over the length of the pilot intervention. These were then extrapolated forward to identify potential healthcare savings.

**Results:**

The SP as an intervention generated the largest cost saving for FAs. The cost variance when FAs participated in the intervention shows there is a direct cost saving of £6,113 or £78.37 per participant over the 5 months of the intervention.

**Conclusions:**

Results suggest there may be a cost saving associated with SP interventions, however caution should be exercised in interpreting the results due to the lack of control group in this study The cost saving were largest for FAs, where the intervention reduced healthcare unit usage as well as actual and inferred impact on associated healthcare costs. This suggests that in practice to generate the maximum cost benefit SP interventions could be targeted at FAs.

## Introduction

The health and social care budget in Wales is almost 50% of the devolved budget (1). In Wales, the number of people aged 65 and over is projected to increase by 37% in the next 20 years (2). Poor health is linked to social and economic disadvantage, resulting in health inequalities (3). Wales has the highest rates of long-term limiting illness in the UK, the most expensive facet of NHS care (4) and there is a more prescribed medication in deprived areas coupled with a higher prevalence for mental health problems (5). The Welsh Government has put in place a number of legislations recognizing the role of non-clinical support as a key part of a social model of health and wellbeing. These are the Wellbeing of Future Generations (Wales) (6) and Social Services and Wellbeing (Wales) Acts (7) and a National Primary Care Plan (8).

### Social prescribing (SP)

It has been argued that psychosocial issues and long-term conditions can be better managed in the community (9). Social prescribing (SP) is “a mechanism for linking patients with non-medical sources of support within the community” such as charities, the voluntary sector, and community groups (10), all of which can offer an alternative to the traditional medical models and reduce the burden on the NHS. SP is a current priority for all of the devolved Nations. The Welsh Government “Social Prescription Model” aims to improve the mental health support available to people with low to moderate mental health issues. In England SP is referenced in the long-term plan with social prescribers or “link workers” embedded in primary care networks (11). Social prescribing interventions are often targeted at people in socioeconomically deprived areas, broadening the options available for primary care when patients present with needs related to wider social determinants of health (12). Our research has found that these patients are often the most frequent GP attenders with the greatest complex needs (13).

There are multiple benefits for patients accessing social prescribing, including increased self-esteem, confidence and sense of control, empowerment, improved psychological and mental wellbeing and mood, and reduced symptoms of anxiety and depression. In addition to this, patients are able to become more active in managing their conditions, resulting in less reliance on the NHS. This is particularly the case for marginalized groups such as mental health service-users and older adults at risk of social isolation (14, 15). Accessing a broad range of community-based services can also help patients' self-manage long-term chronic conditions and reduce health inequalities, particularly for vulnerable and socially deprived groups who face barriers to accessing appropriate health services (16, 17).

Evidence examining the impact of social prescribing on the health service is limited, and the research that is currently available has found mixed results. For example, whilst some evaluations of social prescribing schemes have found reductions in A&E attendance and demand for GP services (18), others have generated little evidence of positive impact. Conversely, another study found no significant difference in the frequency of GP visits or the number of repeat prescriptions before and after completion of a social prescribing intervention (19).

Whilst there is a growing evidence base of the positive health and wellbeing outcomes of social prescribing. The evidence for economic impact is mixed. This study aims to evaluate the cost variances based on healthcare unit usage before and after a pilot social prescribing intervention.

## Methods

The data for this economic evaluation of a pilot SP intervention was collected over 5 months across four GP practices located in areas of high deprivation in Wales.

Patients were referred to two social prescribers by GPs at the practices. No strict inclusion and exclusion criteria were given regarding which patients to refer. Rather, this was left to be determined according to the discretion and clinical judgement of the GPs. The pilot was funded by the Welsh Government to test a social prescription model.

The two social prescribers involved in the pilot saw a total of 78 patients over the 5 months of the intervention *via* face to face appointments. This cohort were subdivided into two groups: frequent non-attenders (*n* = 57) and frequent attenders (*n* = 21). Frequent attenders (FA) are expected to have on average 30 face-to-face GP consultations over 2 years (20). Using this criteria and applying it to the sample in this study FAs are defined as participants who had attended 15 or more GP appointments over the previous 12 months. Healthcare organizations are looking for ways to simultaneously decrease costs and improve patient outcomes (21). FAs are the most prolific users of healthcare resources however evidence suggests interventions targeted at this population yield positive outcomes for these patients (22). Thus we wanted to understand if there was a greater cost saving for this group of patients compared to standard usage.

Referring condition and routine clinical data; GP appointments, current condition, and details of any prescribed medication was extracted from Practice IT systems for each participant 12 months prior to and at the end of the intervention. Data was anonymized before extraction with unique ID codes. A cost variance analysis was undertaken.

## Results

The referring conditions are displayed in Table 1. The largest proportion of participants (33%) were referred due to low mood and isolation difficulties, followed by anxiety and associated social issues (31%), depression and social difficulties (22%) and finally stress and associated social issues (14%).

**Table 1:** Referring condition.

**Conditions by categories**	***N***	**Percentage**
Anxiety and social issues	24	31%
Depression and social difficulties	17	22%
Low mood and isolation	27	33%
Stress and social issues	10	14%
Total	78	100%

The total number of GP appointments and prescriptions dispensed for all the 78 participants are presented in Table 2. Results are presented for 12 pre-intervention, monthly average per participant pre-intervention, total of all participants over the 5 months of the intervention along with the variance in healthcare unit usage. Results indicate that there is a reduction in GP appointments by 4.74 per participant.

**Table 2:** The number of GP appointments and prescriptions dispensed.

	***N***	**Total for 12 months pre-intervention for all participants**	**Average per participant per annum pre-intervention**	**Total for all participants over 5 months of intervention**	**Average per participant over 5 months of intervention**	**Variance in healthcare unit usage**
GP appointments	78	979	12.55	370	4.74	91
Prescriptions dispensed	78	342	4.38	130	1.67	30

This variance in the number of GP appointments pre and during intervention and if extrapolated over the next 12 months has a projected saving of ~£4,823 per annum when applying the suggested unit costings of GP cost per clinic consultation lasting 17.2 min, which is £53 (23). A similar trend was identified for prescriptions dispensed with associated cost savings of £1,290 per annum, based on prescription costs of £43 per consultation (net ingredient cost) when applying the suggested unit costings (23). Examination of the cost variance when clients received the social prescribing intervention shows that there was an overall direct cost saving of £6,113 or £78.37 per participant. Extrapolating this variance over a 12-month period, should circumstances remained constant there is a likely cost saving of £78.20 per participant or a total of £6,099.60 per annum. This is compared with healthcare unit usage in the preceding 12-month period and represents the effects of participating in the SP intervention. Healthcare unit usage and costs outlined in Table 3.

**Table 3:** Healthcare unit costs for entire cohort.

	***N***	**Total for 12 months pre-intervention for all participants**	**Total cost per annum pre-intervention for all participants**	**Total monthly average cost**	**Cost for all participants over 5 months of intervention**	**Total monthly average cost**	**Average cost per participant over 5 months of intervention**	**Projected costs per participant over 12 months post-intervention**	**Projected costs for all participants over 12 months post-intervention**
GP appointments	78	979	£51,887	£4,323	£19,610	£3,922	£251.22	£602.92	47,027
Prescriptions dispensed	78	342	£14,706	£1,225	£5,590	£1,118	£71.81	£172.34	13,442
Total	78	1,321	£66,593	£5,548	£25,200	£5,040	£323.03	£775.26	60,469

### Frequent non-attenders (FNA)

The FNA subgroup of the sample consisted of 57 participants all had attended <15 GP consultations in the previous 12 months. When monthly averages of healthcare unit usage and costs are examined per FNA, there is a slight upward trend in cost average per month related to healthcare unit usage. Results suggest an average cost of £47.35 per FNA in the previous 12 months compared to a monthly average of £53.44 over the 5 months of the intervention. Once costs are extrapolated and inferred for the 12 months following the intervention, there is an increase in costs from £568.24 to £635.40 per annum and a projected increase in costs of £67.16 per frequent non-attender or £3,828 for all 57 FNAs. These estimates suggest that the intervention is not as effective and efficient in reducing healthcare unit consumption for the FNA participants and indicates that, following an SP intervention, they are likely to increase their healthcare unit usage and the associated costs of this.

### Frequent attenders (FA)

For comparison the healthcare unit usage (GP appointments and number of prescriptions) for the 21 FAs pre intervention was examined and indicated that they had a total of 535 face-to-face GP consultations in the previous 12 months, equating to a monthly average of 44 appointments or FA's an average of just over 25 appointments per person. Thus over the 5 months of the intervention there is an overall direct cost saving of £6,113 or £78.37 per FA there is a significant reduction in GP appointments and prescriptions dispensed.

Application of the recommended unit costings of GP appointments (23), and a variance in GP appointments would have a projected total cost difference of ~£8,109 or £1,621.80 per month or £77.22 over the 5 months of the intervention or £497 per FA per annum. A similar downward trend was identified with prescriptions dispensed pre and during the intervention with associated cost difference of £1,677 when applying the suggested unit costings (23).

Inferred costs over a 12-month period post-intervention based on the reduction in healthcare usage and should all things remain equal the likelihood there could be a cost of a reduction to £1,154 per FA per annum. When compared with costs per FA in the previous 12 months of £1,651 per annum per FA there is a reduction of £497 per FA per annum. Hence„ should all things remain equal in the subsequent 12 months post-intervention there is inferred cost difference, which is total cost for all FA over 12 months minus the projected healthcare usage cost in the next 12 months (£34,676–£24,247 = £10,429) as outlined in Table 4.

**Table 4:** Pre and post-intervention cost analysis for FA.

	***N***	**Total for 12 months pre-intervention for FA**	**Total cost per annum pre-intervention for all FA**	**Total monthly average cost**	**Average per FA per annum**	**Average cost per FA over 5 months of intervention**	**Projected costs per FA over 12 months post-intervention**	**Projected costs for all FA over 12 months post-intervention**
GP appointments	21	535	£28,355	£2,363	£1,350	£401.28	£963	20,223
Prescriptions dispensed	21	147	£6,321	£526.75	£301	£79.85	£191.64	4,024
Total	21	682	£34,676	£2,889.75	£1,651	£481.13	£1154.64	24,247

## Discussion

The pilot SP intervention in this study was delivered over 5 months and involved a total of 78 participants. In order to examine the effect of the intervention and estimate its impact, participants were divided into two subgroups FAs and FNAs. Associated costs were then calculated based on healthcare unit usage defined as GP consultations and prescriptions dispensed.

Results indicate for all of the patients who participated in the intervention there was a direct cost saving of £78.37 per participant or £6,113 for the total cohort over the 5 months of the intervention. Extrapolating these reduced costs over a 12-month period shows that there could be potential cost saving for the entire cohort (*n* = 78), of £6,099.60 or £78.20 per participant in reduced healthcare unit usage per annum.

Conversely, when the cohort were subdivided into two distinct groups, FAs and FNAs, results indicated variances between the two. Estimation of monthly average costs for each FNAs while on the intervention and inferred for the following 12 months, the estimates suggest that per annum there would be an increase in costs. However, among the FA's group (*n* = 21) results suggest that the intervention had a considerable influence on reducing healthcare unit usage and costs. Twelve month projections taking account of potential changes in unit of healthcare usage suggests that, should all things remain equal, there should be a cost reduction of £497 per FA patient per annum. Hence, should all things remain equal in the following 12 months post-intervention there would be a contingent cost reduction of £10,429 for all of the FA's as a result of reduced healthcare unit usage.

One possible explanation for the results is improved Patient Activation (PA). PA has become a popular construct in public health and management of long-term conditions in recent years. PA is defined as knowledge, “skills and confidence a person has in managing their own health and health care” (24). Having the skills and knowledge of one's own conditions can lead to a better level of activation (25, 26) and having higher levels of PA positively contributes to patients' management of health conditions (27).

Patient activation is also a suggested key mechanism in ensuring the effectiveness of SP interventions in achieving improved outcomes for patients (28). This has also been found in qualitative evaluations of SP interventions (29, 30). SP emphasizes patient choices and empowerment by using a range of therapeutic and behavioral change techniques such as coaching, motivational interviewing and empathetic listening skills in order to create the core conditions needed to promote behavior change (31). This is a key feature that supports patients in their journey toward activation and behavior change. SP has also been shown to significantly improve PA scores for over 50s with long terms conditions, yet no economic or healthcare utilization benefit was identified in this study (31). Hence, it can be hypothesized FAs increased their PA through taking part in the intervention resulting in better self-management of their presenting health conditions leading to less healthcare usage, reduced GP appointments and prescriptions. Conversely findings for the NFAs a marginal increase in health care unit usage and associated costs can still be explained by increased PA in this group of patients. If those patients become more activated, they may visit the GP more as a way of actively managing their health condition.

This economic evaluation of this pilot SP intervention demonstrated there are cost savings particularly for FAs taking part in a SP intervention. Extrapolation of estimates and forward projection indicates that the SP intervention in this study could potentially yield greater cost savings and benefits if delivered over a longer period, particularly when aimed at specific cohorts. The cost information may be of use to decision makers in determining the allocation of finite resources, whilst also providing information on the benefits of alternative non-clinical services that have both health and wellbeing effects and a positive impact on resource use.

Whilst FAs may have the largest number of needs and represent the biggest burden on GP practices, they are also the group that produced the biggest savings in the current study, both in terms of reduced GP appointments and demand on practice staff time. These patients' issues require more than a “quick fix,” and they require a much more person-centered approach. This could be a challenge for social prescribers, who may not be trained nor have the competence to deal with such complex issues. Further research should investigating if different SP delivery lengths are more appropriate for FAs and explore “dose–response” relationship “minimum duration for maximum benefit” to maximize patient outcomes and the cost benefits.

Although it is widely acknowledged that social and economic factors affect health outcomes, and there is limited evidence on the economic benefits of SP intervention addressing public health needs. The cost analysis findings in the present pilot SP intervention are consistent with previous studies which have demonstrated a cost reduction following SP as a result of fewer GP appointments and reduced use of prescription medication (32). Furthermore, social prescribing alleviates immediate time, infrastructural and monetary pressures from GPs, the NHS and other parties involved in primary care (33). Evidence also suggests that social prescribing positively impacts upon GP time and as such has a cost saving. The freeing up of GP capacity can have positive effects on patient safety and staff morale, along with reductions in stress (34). Ultimately, taking alternative approaches to meet the needs required in primary care can reduce pressure for GP appointments and services (35), and future studies should undertake a comprehensive cost benefit evaluation which would allow for a more objective assessment of the value of SP and explore whether there is an association with increased PA and positive health outcomes for patients.

### Limitations and implications

This was a 5-month pilot, which was determined by limited funding scheme rather than the clinical need of patients or the available evidence regarding the most effective length of time to run a social prescribing intervention. Because of this, there was not enough time for the programme to be embedded within all practices, leading to peaks and troughs in referrals as practices got more engaged. It is recommended that future pilots are extended to at least 12 months to allow the intervention to fully embed in GP practices. Due to the necessity of needing to provide the intervention to all eligible patients there was no control group hence conclusions can only be tentative. Based on the need to evaluate the intervention *in situ* and the reliance on practice staff to add the correct information to the system, and to download the relevant information for analysis, this resulted in incomplete data sets for intervention participants. Thus confidence intervals around estimates could not be conducted.

Data quality is an ongoing challenge in “real-world” cost analysis where researchers are reliant on doing *post-hoc* evaluations on the best data available (36). As a result we were unable to gather detailed data and other quality indicators and cost analysis was conducted at the end of the intervention. Due to these limitations researchers were not able develop a data collection protocol prior to the intervention being conducted hence the criteria for consolidated health economic evaluation reporting standards (CHEERS) could not be met (37). Future studies should where possible ensure that economic evaluation quality reporting standards such as CHEER are used in study development and set up prior to conducting the evaluation to improve the data quality and reporting. Despite these limitations even in the relatively short time of the intervention delivery the data does appear to demonstrate that there is a reduction in healthcare unit usage and a cost saving for FAs. However, without further data it is difficult to know whether the frequent attendance is temporal rather than persistent and continuous over time. A longer timeframe would mean that more patients could be referred to the intervention, allowing more data to be collected and the testing of the assumption that it does indeed improve patient outcomes and reduce the frequency of attendance. Finally, controlled trials are also needed to observe causality, and to explore whether the outcomes found in the current study are replicable.

## Data availability statement

The datasets presented in this article are not readily available because the processed data required to reproduce the above findings cannot be shared at this time due to ethical reasons. Requests to access the datasets should be directed to crj10@leicester.ac.uk.

## Ethics statement

Secondary analysis of de-identified data was undertaken. Ethics approval was granted by Cardiff University Ethics Committee. The patients/participants provided their written informed consent to participate in this study.

## Author contributions

CRJ and ML: conceptualization, methodology, and writing plus review and editing. CRJ: resources. ML: formal analysis. All authors have read and agreed to the published version of the manuscript.

## Funding

This pilot project was funded through the Innovate to Save (I2S) scheme, which is funded by the Welsh Government. The I2S fund provides financial and non-financial support to Welsh public services in order to prototype and test innovations to improve services. The I2S fund operates alongside the Welsh Government Invest to Save fund, a repayable, interest-free, loan which successful I2S projects can apply for.

## Conflict of interest

The authors declare that the research was conducted in the absence of any commercial or financial relationships that could be construed as a potential conflict of interest.

## Publisher's note

All claims expressed in this article are solely those of the authors and do not necessarily represent those of their affiliated organizations, or those of the publisher, the editors and the reviewers. Any product that may be evaluated in this article, or claim that may be made by its manufacturer, is not guaranteed or endorsed by the publisher.
